# Comparative Study on Trunk Range of Motion in Land Balance Tests and Snow Skiing: Implications for Para‐Alpine Sit‐Ski Performance: A Cross Sectional Study

**DOI:** 10.1002/hsr2.70858

**Published:** 2025-06-11

**Authors:** Yusuke Ishige, Yuki Inaba, Noriko Hakamada, Akio Kobayashi, Shinsuke Yoshioka

**Affiliations:** ^1^ Department of Sport Science Japan Institute of Sports Sciences, Japan High Performance Sport Center Kita‐ku Tokyo Japan; ^2^ Department of Orthopaedic Surgery Shiraniwa Hospital Ikoma Nara Japan; ^3^ Graduate School of Arts and Sciences The University of Tokyo Meguro‐ku Tokyo Japan

**Keywords:** centrifugal force, Para Alpine Skiing, sitting category, the Board Test, trunk range of motion

## Abstract

**Background and Aims:**

In the present study, we examined the effect of trunk range of motion (ROM) on turn performance in Para Alpine Skiing by comparing the ROM of the trunk on land with that of the trunk on snow in different classes. We attempted to clarify the relationship between impairment tests and athletic performance by examining the relationship between centripetal acceleration during skiing and trunk ROM, which is difficult to reproduce during athletic activities on land.

**Methods:**

Six male and four female skiers from sitting classes participated in this study. MRI images of the trunk and thigh were obtained. The ROM of the trunk was measured using the motion capture method during the Board Test on land and freeskiing on snow.

**Results:**

The ROM of the trunk in the Board Test increased with a lower degree of disability. However, there was no relationship between the ROM of the trunk on snow and the degree of disability. A number of lateral flexion angles during turns exceeded the ROM of the trunk on land. The magnitude of centrifugal force did not depend on the degree of impairment. The relationship between the centrifugal force and the lateral flexion angle was linear.

**Conclusion:**

The Board Test is an appropriate impairment test. During the turn, the body is passively moved because of the centrifugal force acting on it, a value that significantly exceeds the ROM of the trunk on land. The ROM of the trunk on snow was independent of the degree of disability.

## 1 Introduction

Para Alpine Skiing has a short history, with SL and GS events taking place at the first Winter Paralympic Games in Örnsköldsvik in 1976. In Para Alpine Skiing, there are separate sport classes for standing, sitting, and visual impairment. Sit‐skiing became a medal event at the 1998 Paralympic Games in Nagano. In Para Alpine Skiing, ~440 athletes are registered for the 2023–2024 season. The number of athletes in Para Alpine Skiing is the largest among the Winter Paralympic sports categories, along with wheelchair curling. In recent years, Paralympic sports have become competitive sports, with races held on the same slopes as able‐bodied athletes.

Despite IPC's recommendation for evidence‐based classification [1, 2], there is a lack of research on para‐alpine areas to provide evidence for classification. Classification is sport‐specific because impairment affects the ability to perform different sports to varying extents. In particular, the relationship between impairment tests and athletic performance remains unclear. Thus, the performance determinants have not been clarified. This is especially true for the para‐alpine regions. This study aimed to clarify the performance determinants of para‐alpine sitting category skiers, which will be the first step toward evidence‐based classification. While research on evidence‐based classification has been conducted mainly in summer sports [3, 4, 5, 6, 7, 8], only a few studies have been conducted on Para Alpine Skiing [9, 10]. This is presumably because of the difficulty in conducting experiments on snow and the extreme difficulty in securing the subjects required for statistical processing. In this study, we examined the sitting category skiers whose lower body function is restricted by the disability; however, the equipment shares the function of the lower limbs. If we ignore the performance of the equipment, it is the trunk that controls the turn; therefore, we thought it would be easier to clarify the relationship between the impairment tests and athletic performance than in the other categories.

Various factors define the competitive performance of sit‐skiers, such as the turn technique, physical strength, and psychological factors; however, one of them is the turn technique of controlling the ski with the trunk. Since sit‐skiers control their ski with their trunk, it is assumed that any impairment of the trunk will affect their performance while skiing. Therefore, we inferred that there is a relationship between trunk function and performance in alpine sit‐skiing because these classifications are based on the residual function of the trunk. Trunk function was assessed using magnetic resonance imaging (MRI) images as an interpolation of impairment tests.

Controlling the edge angle by angulation is the most important mechanical principle in alpine skiing [11]. As angulation during sit‐skiing is controlled by lateral flexion and rotation of the trunk, its limitation is considered to have a significant impact on sit‐skiing. Hip angulation angles approaching 40° have been reported in slalom races with able‐bodied skiers [12]. This angle is nearly identical to the full range of lateral flexion in the thoracic and lumbar spine regions [13]. This means that even those with unrestricted lateral flexion of the trunk have no extra ROM when sit‐skiing [14]. This suggests that when maximum lateral flexion of the trunk during skiing is limited according to the level of disability, the resulting angulation is also limited. Because the degree of angulation, in combination with the skier's body's center of gravity tilt, determines the edging angle and affects the minimum radius of the carving turn [15], insufficient angulation is expected to be detrimental to competition. As a general rule of thumb, the angulation angle increases as centripetal acceleration increases. The centripetal acceleration takes on larger values as the radius of curvature of the turn decreases and as the speed of the turn increases. To withstand higher centripetal acceleration, the skier must increase the angulation angle. In the present study, we examined the effect of the ROM of the trunk on turn performance by comparing the ROM of the trunk on land with the ROM of the trunk on snow in different classes. We attempted to clarify the relationship between the impairment test and athletic performance by examining the relationship between centripetal acceleration during skiing and trunk function (ROM), which is difficult to reproduce during athletic activities on land.

## 2 Materials and Methods

Nine sit‐ski athletes (five males, four females; average age 36.3 ± 10.4 years, average arm span 1.67 ± 0.10 m, average weight 56.2 ± 14.5 kg) who were members of the Paralympic National Alpine Ski Team and one male athlete who had previously been a member of the national team participated. Information on subject classes, years from injury and number of Paralympic Games appearances is summarized in Table 1. The impairments in the LW10‐2 skier, LW11 class skiers, and LW12‐1 skier were spinal cord disorders; LW12‐2 skiers had both lower extremities amputated at the thigh or shank due to a traumatic accident. The subjects provided written informed consent to participate in the study, which was approved by the ethics committee of the authors' institution (Approval No. H30‐0047).

**Table 1:** Participant characteristics.

Participant	Sex	Years from injury	Class	Number of Paralympic Games appearances
1	Female	21	LW10‐2	3
2	Male	28	LW11	1
3	Male	25	LW11	6
4	Male	27	LW11	5
5	Male	18	LW11	3
6	Female	13	LW11	1
7	Male	13	LW11 (review)	0
8	Female	40	LW12‐1	1
9	Male	27	LW12‐2	5
10	Female	44	LW12‐2	4

**Figure 1:**
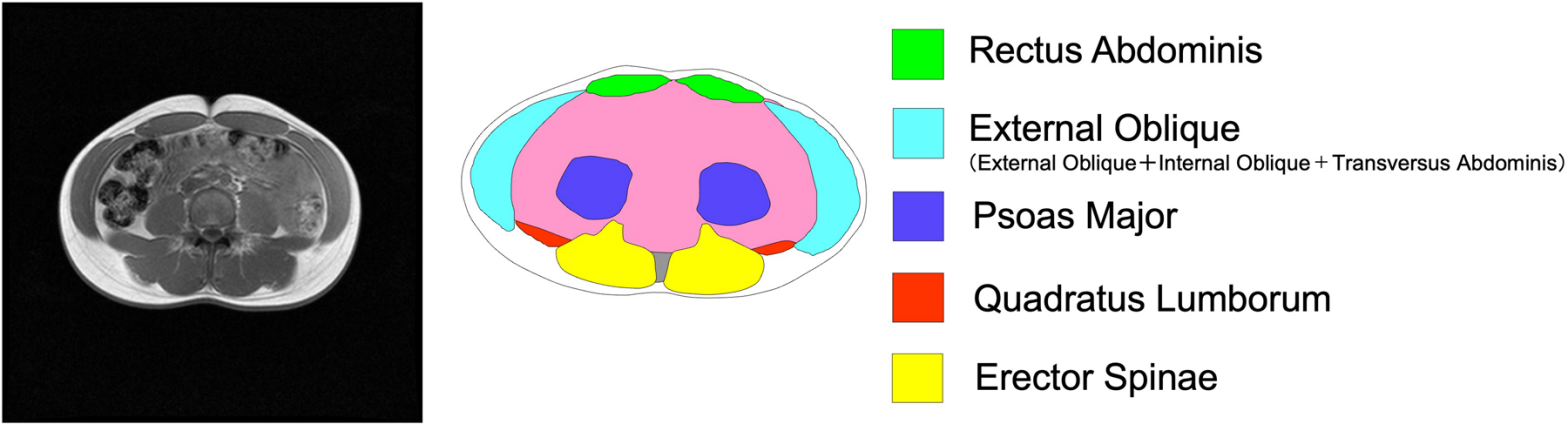
The left figure shows the typical MR imaging scans at the top of the pelvis: Jacoby Line of one subject. The right figure shows the general classification of the targeted muscles (rectus abdominis, external oblique, psoas major, quadratus lumborum, erector spinae).

A 3‐T superconducting MRI device (Magnetom Verio; Siemens Healthineers, Erlangen, Germany) was used to obtain MRI to measure the cross‐sectional area (CSA) of the muscles using a body coil. The athletes were placed in the supine position. For the thigh, localization images were obtained from three anatomical planes (sagittal, coronal, and transverse). Next, transverse fast spin‐echo images (repetition time, 500 ms; echo time: 8.2 ms; matrix, 256 × 256; field of view, 240 mm; thickness, 10 mm) were obtained halfway between the trochanter major and the tuberculum intercondylaris. For the trunk, localization images were obtained from the three anatomical planes (sagittal, coronal, transverse), and then transverse gradient echo image (repetition time 90 ms, echo time 2.46 ms, flip angle 55°, matrix 256 × 256, field of view 380 mm, thickness 10 mm) was obtained at Jacoby's line, which connects on the superior border of the iliac fossas. To avoid motion artifacts attributable to breathing, MRI of the trunk was performed with the subject holding his breath at the inspiratory position. CSAs for the rectus abdominis, external oblique abdominis, psoas major, quadratus lumborum, and erector spinae muscles were computed by tracing the obtained images (Figure 1). The total muscle CSA was computed as the sum of the CSAs of those identified muscles.

**Figure 2:**
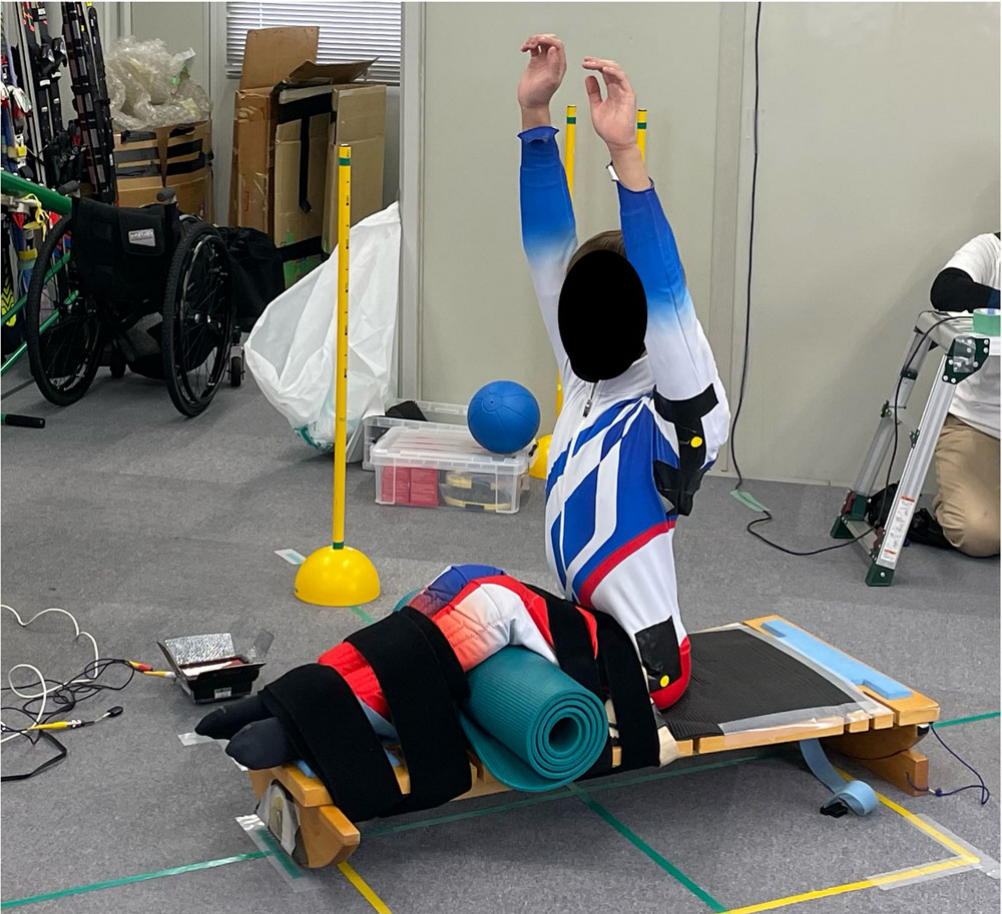
Board Test measurements.

The Board Test was conducted in accordance with FIS rules (Figure 2; [16]). The Board Test was selected because the test content was specific to athletes competing in a sitting position.

Test 1: Upper extremity strength, range of movement, and function testing while sitting straight on the testing board and performing arm movements (shoulder abduction, anteflexion, retroflexion, elbow flexion, and extension). Observe how this influences the sitting balance. The test board was then blocked.

Test 2: Sitting balance in the sagittal plane (flexion). Sitting with the hands behind the neck, flex forward as much as possible at the waist. The trunk was then extended and lifted to a position of 45° forward flexion. Hold the position, keeping the hands behind the neck. The test board was blocked.

Test 3: Sitting balance in the sagittal plane (extension). The arms folded over the chest and extended back to hold a 45° backward extension. The test board was blocked.

Test 4: Sitting balance in the sagittal and frontal planes (rotation). Free rotation of the trunk in the sitting position with the arms fully abducted. The test board was blocked.

Test 5: Sitting balance in the frontal and sagittal planes to test trunk and pelvis stability. A 1 kg ball was placed beside the athlete's hip at the level of the testing board. The athlete picked up the ball with both hands and lifted the ball above the head to place it beside the hip on the opposite side of the testing board. This is repeated in the other directions. The test board was blocked.

Test 6: Sitting balance in the frontal plane (tilting).

The Board Test was evaluated by a member of the FIS Classification Panel. For each Board Test, one of the following scores was allocated.

0 = No Function, test impossible

1 = Weak or poor function

2 = Fair function

3 = Normal function

The total score of all six parts of the Board Test was used to allocate the Sport Class.

To evaluate the angle of the subject's trunk during the Board Test, motion analysis was performed using three digital video cameras (30 fps) placed at the top of the subject's head, on the back, and on the left side of the subject's body to simultaneously capture the subject's trunk posture in sagittal, frontal, and horizontal planes. Based on the anatomical landmark points (left and right acromion points, left greater trochanter point, and left and right superior posterior iliac spine points) affixed to each part of the subject's body, the trunk angle was calculated using the 2D DLT method with dedicated image‐processing software (Frame‐DIAS 6, DKH, Tokyo). The angle of the trunk is defined as follows:

*Trunk flexion/extension angle*: Angle between the gravity line and the line of the trunk (line connecting the acromion point and the greater trochanter point), viewed from the sagittal plane (direction of extension is positive).

*Trunk rotation angle*: Angle between the gravity line and the line connecting the right and left acromion points, viewed from above (right rotation (left arm front) is positive).

*Left‐right lateral flexion angle*: Angle between the horizontal line and the line connecting the left and right superior posterior iliac spine points, viewed from the back (right tilt is positive).

Field measurements were performed in February (one athlete) and March (nine athletes) in the Sugadaira ski area, Nagano Prefecture, Japan, as the athletes were in the competitive season. This ski area was chosen because the weather is stable in winter and the snow conditions are also stable. With the exception of one athlete who was measured in February, we tried to minimize the effects of the weather and snow conditions by measuring them during the same period (March 19–28, average minimum/maximum temperature −1.3°/8.7° (Japan Meteorological Agency)). The snow conditions in both February and March were within the normal training environment for each athlete. A Pine Beak Ski Resort Go no Hara course was used. The average (maximum) slope angle, vertical drop, and course length were 12° (18°), 103 m, and 500 m. This course was chosen because it has a uniform slope and is therefore suitable for measuring uniform and stable turns. Snow conditions were hard in the early morning, then gradually softened as the temperature rose during the day. All field measurements were performed between 8 a.m. and 12 p.m., when the snow conditions were stable. Skiers performed two runs of freeskiing with their own slalom ski and sit‐ski, and the second run was used for further analysis.

The ROM of the trunk on snow was measured using the motion capture method with inertial measurement units (IMUs) [14, 17]. The key to this measurement method is to accurately determine the offset of the gyro sensor contained in each IMU. Immediately before each trial, the athlete took a stationary posture for ~40 s. The average value of each gyro sensor during the stationary period was determined as the offset value of that sensor for that trial. The initial posture of each IMU was calculated from the data of the acceleration sensor contained in the IMU (i.e., the gravitational acceleration). The initial posture of the athlete was obtained from the photographs taken from the side and from the rear. The relative posture of the IMU with respect to each body segment of the athlete was calculated from their initial postures. The above calibration was performed immediately before each trial. Signals from IMUs were sampled at 1000 Hz. Kinematic data were low‐pass filtered at a cut‐off frequency of 10 Hz with a fourth‐order Butterworth filter. The cut‐off frequency was determined using a residual analysis [18].

The flexion/extension, lateral flexion, and rotation about the long axis of the trunk were expressed using the Euler angle. The order of rotation was determined in accordance with the recommendation of Grood and Suntay [19].

The centripetal acceleration was calculated by subtracting the gravitational acceleration from the acceleration data obtained from the IMU attached to the ski seat (corresponding to the pelvis). In addition to the gravitational and centripetal acceleration, the acceleration data include a component of vibration that arises from the collision between the ski and the fine bumps of the snow surface (higher frequency component). The measured acceleration was low‐pass filtered at a cut‐off frequency of 2 Hz. The cut‐off frequency was determined based on the frequency components of the acceleration data obtained by the Fourier transformation.

In the analysis, each run was divided into right and left turns. The moment when the centripetal acceleration became zero was defined as the switching time of the right and left turns. Ten consecutive turns (five right and five left turns) during one run were selected based on the consistency in the point of turn time. For all athletes, ten turns from the fifth or sixth turn to the fourteenth or fifteenth turn were selected. The average of the five turns was used as the on‐snow result for each skier.

The collected data were analyzed to determine key statistical parameters. Specifically, we calculated the mean and standard deviation (SD) for the groups of LW11 and LW12 class skiers, and expressed as mean ± SD. Additionally, we computed the 95% confidence interval for the CSA of trunk muscles and identified the maximum and minimum values for the score of the Board Test. For the LW10 athlete, as *n* = 1, we did not perform any statistical tests.

## 3 Results

CSAs for the rectus abdominis, external oblique abdominis, psoas major, quadratus lumborum, erector spinae muscles, and the total muscle CSA (the sum of CSAs of those muscles) are shown in Table 2. No CSA was identified in the psoas major, quadratus lumborum, or erector spinae muscles of the LW10‐2 skier and one LW11 skier [20]. The differences in CSAs of all muscles between the classes tend to be small except that the total muscle CSA in the LW10‐2 skier was clearly smaller than that in the LW11 and LW12 skiers.

**Table 2:** Cross‐sectional areas (CSAs) of trunk muscles (rectus abdominis, external oblique, psoas major, quadratus lumborum, erector spinae) at the top of the pelvis: Jacoby Line of skiers.

	Cross‐sectional areas of trunk muscles (cm^2^)				
	LW10‐2	LW11		LW12	
	*n* = 1	Mean ± SD	95% CI	Mean ± SD	95% CI
Rectus abdominis	< 13	13.4 ± 4.2	[10.1, 16.8]	13.3 ± 2.1	[10.9, 15.8]
External oblique	< 35	54.4 ± 17.6	[40.3, 68.5]	40.4 ± 5.3	[34.4, 46.3]
Psoas major	—	9.0 ± 6.7	[3.7, 14,4]	17.4 ± 1.3	[15.9, 19.0]
Quadratus lumborum	—	6.8 ± 4.2	[3.4, 10.2]	13.7 ± 1.9	[11.5, 15.9]
Erector spinae	—	31.7 ± 18.4	[16.9, 46.4]	33.4 ± 4.1	[28.7, 38.0]
Total muscle CSA	< 50	115.3 ± 35.1	[87.2, 143.4]	118.2 ± 9.5	[107.5, 128.9]

*Note:* Mean, standard deviation, and 95% confidence intervals (CI) were calculated for LW11 and LW12. As *n* = 1 for the LW10‐2 skier, detailed values are not shown.

**Figure 3:**
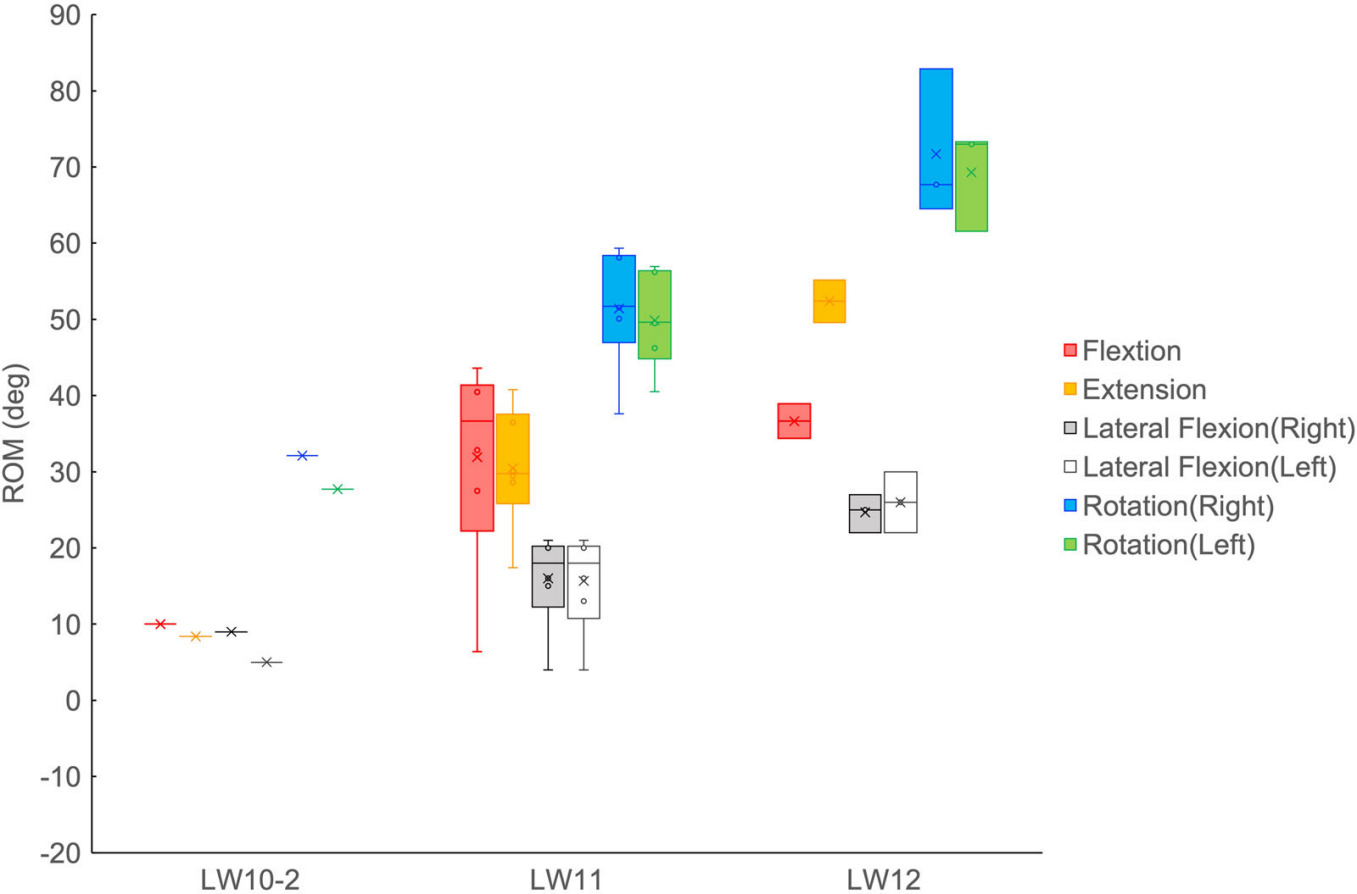
The boxplot of range of motion (ROM) of the trunk in the Board Test. The circles indicate values for each subject, while crosses indicate the mean values of each group and the lines inside the boxes indicate the median values.

The results of the Board Test are listed in Table 3. The total score of the Board Test was the smallest for the LW10‐2 skier while similar results were observed for LW11 and LW12. The ROM of the trunk in the Board Test increased with a lower degree of disability (Figure 3).

**Table 3:** Board Test scores.

		Score of the Board Test			
	LW10‐2	LW11		LW12	
	*n* = 1	Mean ± SD	[Min, Max]	Mean ± SD	[Min, Max]
Test 1	< 3	3.0 ± 0.0	[3, 3]	3.0 ± 0.0	[3, 3]
Test 2	< 3	2.7 ± 0.8	[1, 3]	2.0 ± 1.7	[0, 3]
Test 3	< 3	1.8 ± 1.0	[1, 3]	2.0 ± 1.7	[0, 3]
Test 4	3	3.0 ± 0.0	[3, 3]	3.0 ± 0.0	[3, 3]
Test 5	3	3.0 ± 0.0	[3, 3]	3.0 ± 0.0	[3, 3]
Test 6	< 3	2.5 ± 0.8	[1, 3]	3.0 ± 0.0	[3, 3]
Total	< 12	16.0 ± 2.2	[12, 18]	16.0 ± 3.5	[12, 18]

**Figure 4:**
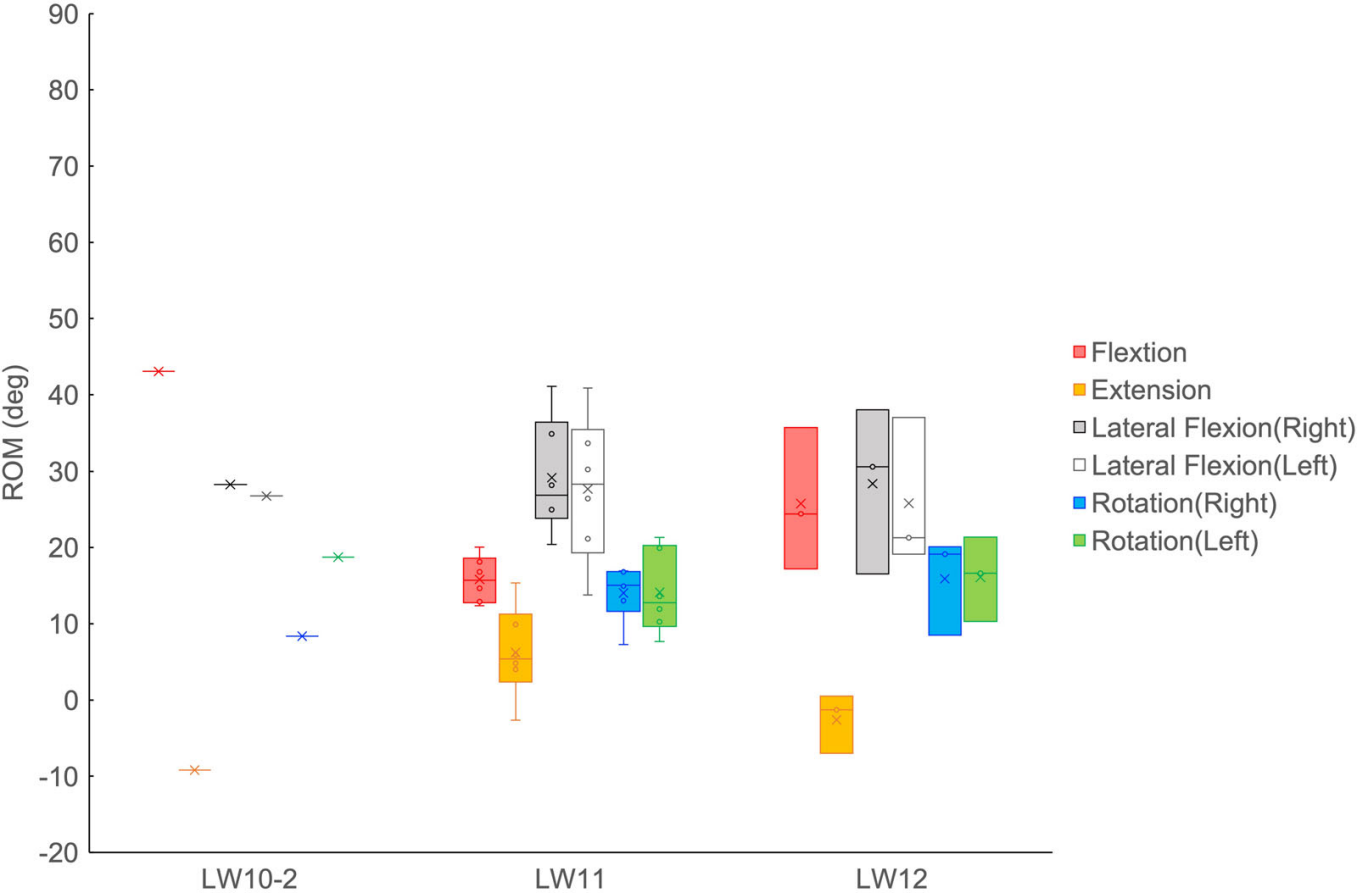
The boxplot of range of motion (ROM) of the trunk on snow. The circles indicate values for each subject, while crosses indicate the mean values of each group and the lines inside the boxes indicate the median values.

**Figure 5:**
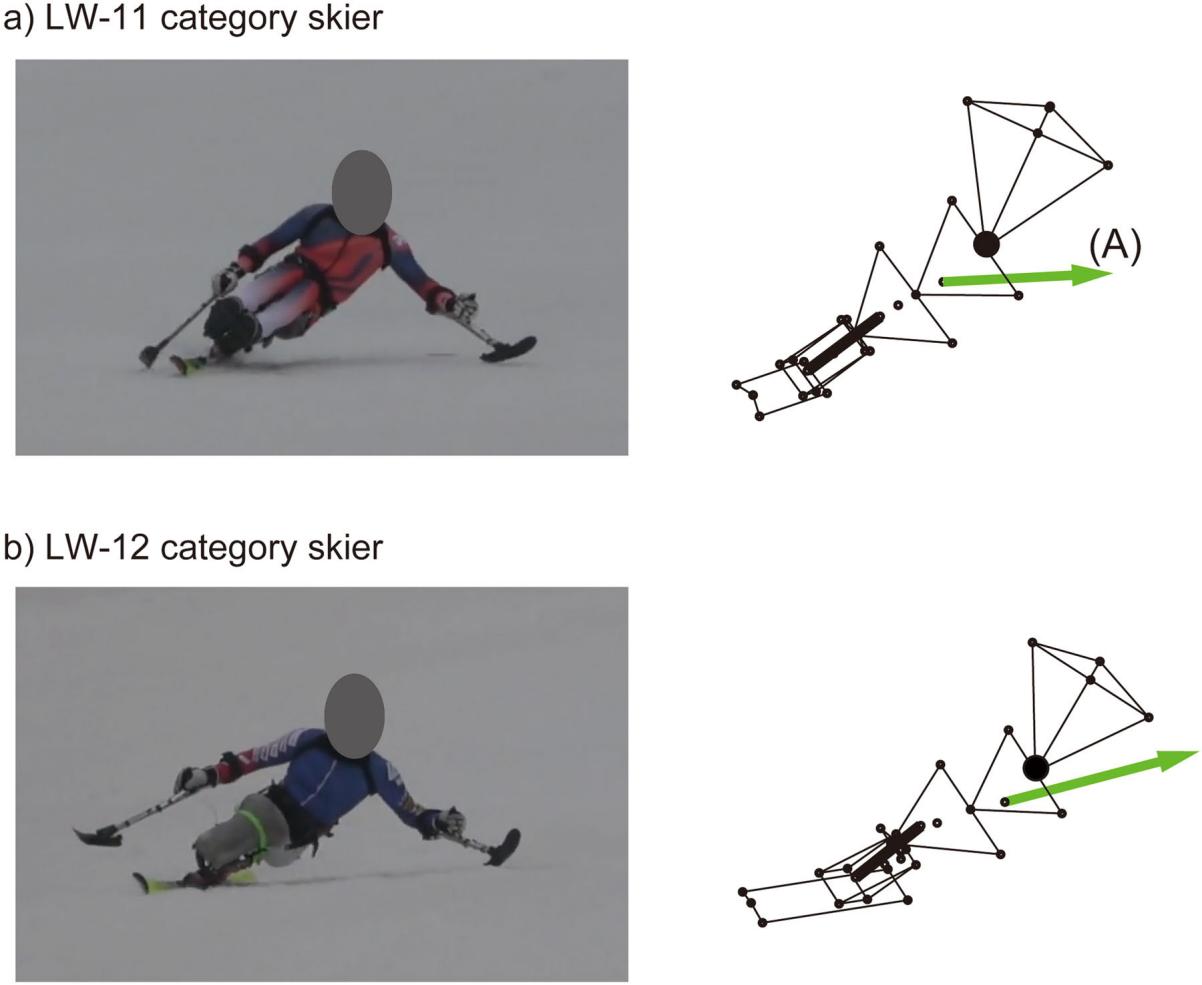
Typical examples of the motion measurement results for (a) LW‐11 and (b) LW‐12 category skiers. The vector (A) indicates the centripetal acceleration. The length of the vector in the stick figure, 1000 mm, indicates an acceleration of 1 G.

**Figure 6:**
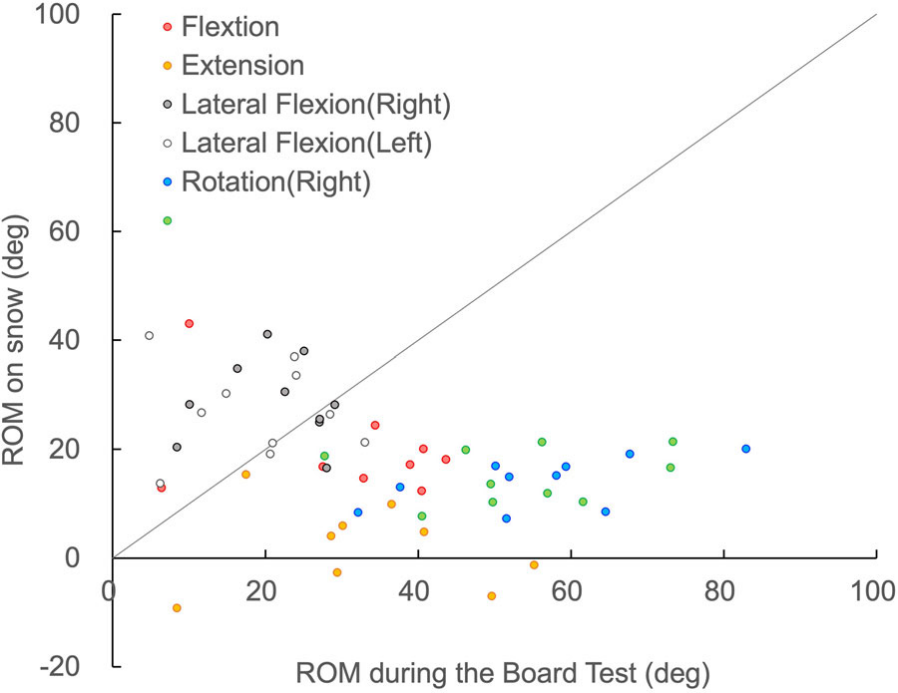
Comparison of trunk range of motion during the Board Test and while skiing.

No clear relationship between the ROM of the trunk on snow and the degree of disability were observed in subjects of all groups (Figures 4 and 5). In other words, the ROM of the trunk on snow was within a certain range (Figure 4). No relationship between the ROM of the trunk on land and on snow was observed. A number of lateral flexion angles during turns exceeded the ROM on land (Figure 6).

**Figure 7:**
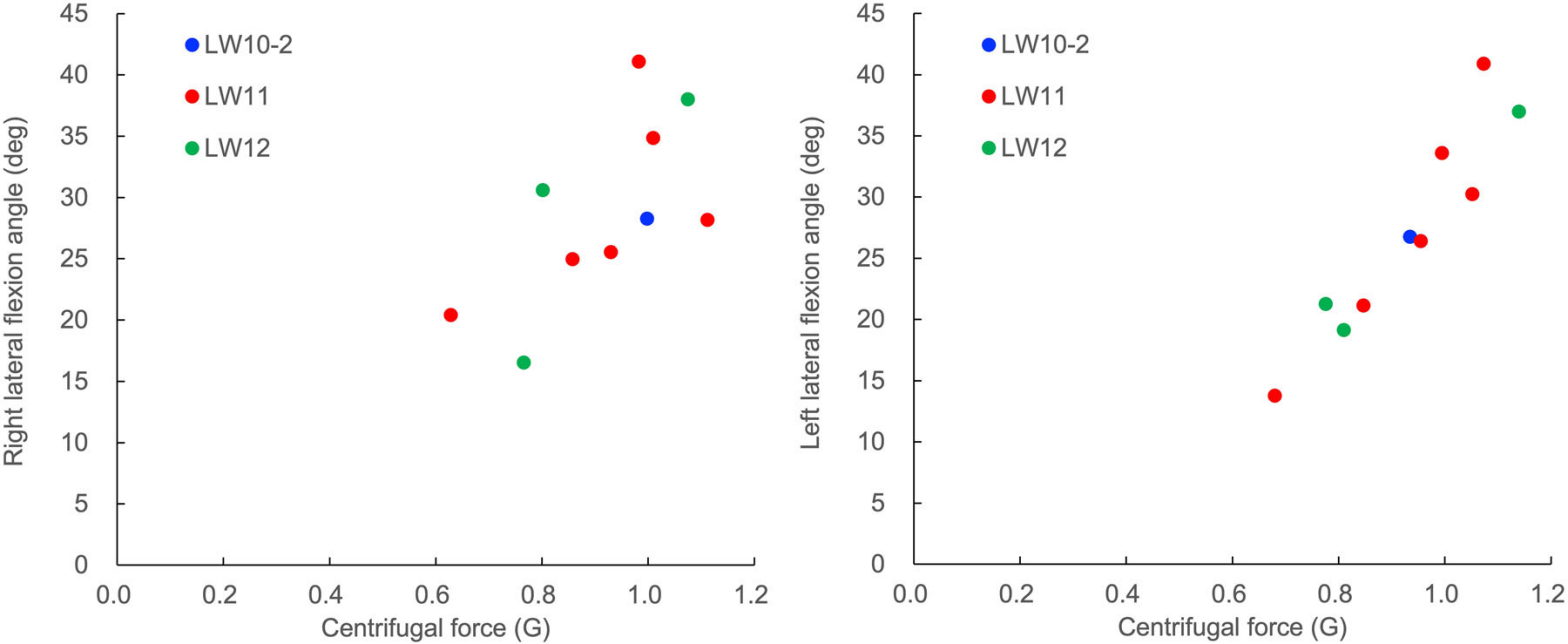
The relationship between centrifugal force and lateral flexion angle. The left figure shows the relationship between the centrifugal force and the lateral flexion angle during the left turn, and the right figure shows the relationship during the right turn.

Differences between right and left trunk lateral flexion and rotation ROM were observed. The right and left trunk lateral flexion ROM differences during Board Test were 4.0° for the LW10‐2 skier, 1.7° ± 2.0° for LW11 skiers, and 1.3° ± 1.5° for LW12 skiers while those on snow were 8.4°, 6.0° ± 4.0°, and 5.7° ± 5.3°, respectively. The right and left trunk rotation ROM differences during Board Test were 4.4° for the LW10‐2 skier, 2.5° ± 1.7° for LW11 skiers, and 6.0° ± 3.4° for LW12 skiers while those on snow were 10.4°, 5.6° ± 3.5°, and 1.9° ± 0.6°, respectively. The magnitude of centrifugal force did not depend on the degree of impairment. The relationship between the centrifugal force and lateral flexion angle was more linear in left lateral flexion (Figure 7).

## 4 Discussion

The CSA of the muscle groups considered to be involved in trunk lateral flexion varied according to muscle location and class. The fact that the total muscle CSA of the LW10‐2 athletes was clearly smaller suggests that differences in total muscle CSA may explain the differences in trunk lateral flexion ROM in the Board Test (Table 2). However, since it is not realistic to make MRI mandatory for all skiers, the results of the MRI should be taken to indicate the validity of the Board Test in this case. The cause of the lack of CSA in the psoas major, quadratus lumborum, and erector spinae muscles in the two subjects was not determined. The decrease in CSA of skeletal muscles after spinal cord injury has been reported in previous studies [21, 22]. Additionally, significantly higher fatty infiltration and greater degree of muscle degeneration in paravertebral extensor muscle have been reported in patients with acute cervical spinal cord injury when compared to healthy controls [23]. However, further investigation is needed to determine whether the trunk muscles of the skiers of the current studies were pathologically degenerated due to atrophy or other reasons, and to determine the musculature of the trunk of seated athletes.

There was no difference between LW11 and LW12 in terms of the mean total score of the Board Test (Table 3). This was due to the fact that one of the three LW12 athletes was unable to perform Tests 2 and 3 due to amputation of both legs of the thigh, resulting in a lower total score for the six tests. The current classification test almost automatically classifies an amputee as LW12‐2 in the case of amputation of both legs; however, this part of the test needs to be considered.

The ROM of the trunk in the Board Test was based on disability level (Figure 3). These results confirm the validity of the impairment tests. Nevertheless, the ROM of the trunk on snow was independent of the level of disability (Figure 4). This can be considered as a characteristic of alpine skiing turns in sit‐skiing, and is important in the context of para‐sports. In Para‐kayaking [24] and Para‐Nordic skiing [25], it has been reported that the degree of trunk impairment is related to the ROM of the trunk during movement, which is different from the results of this study. This difference highlights the sport‐specific nature of trunk control in alpine sit‐skiing.

Many of the lateral flexions during the turn (on snow) exceeded the ROM on land (Figure 6). It is possible that the skier moved passively during the turn. A skier with a small trunk ROM is consequently able to take an outward leaning posture that results in a ROM greater than the ROM that he can actively move; however, this is not voluntarily controlled, and the muscles are likely to be in different states of activity, even though the posture is the same. Bucket seats or belts can also support ROM. In fact, the lateral flexion angle on snow may exceed that on land, and the limited ROM on land does not necessarily have an effect on snow. It is believed that the ROM on snow is the result of passive motion due to centrifugal forces. It has been reported that passive hip flexion and extension occur during GS turns in a LW10‐2 class skier who is unable to flex and extend his/her hip joint voluntarily [14]. It is possible that passive movement is utilized more during sit‐skiing than we had previously thought.

In the slalom race for able‐bodied skiers, the angulation angle reaches about 40° at the hip joint [12]. Since the ROM of lateral flexion of the entire thoracic and lumbar spinal regions is about 40° [13], it is possible to take an angulation using the trunk region. In fact, lateral flexion of up to 40° has been observed in a sit‐ski racer during GS skiing [14]. In healthy adults, no force other than muscle force is required to utilize the entire ROM of lateral flexion in lumbar region [26]. However, in the case of a sit‐skier with the impairment of trunk muscles, such as LW10 and 11 class skiers, a force other than muscle force is required to utilize the entire ROM of the trunk. Therefore, it is thought that centrifugal force is being used. However, this angulation angle is necessarily limited by the upper limit of the flexibility of the trunk, and in the case of an athlete with a limited flexibility, it is expected that the turn performance will be limited by it, regardless of whether centrifugal force is being used or not.

The premise is that if the ROM of the trunk that can be actively moved on land is limited, the angulation angles may be limited on snow (especially lateral flexion and rotation). However, our results refute this conclusion. The present results show that factors other than the ROM of the trunk in the Board Test affect turn performance on snow.

The centrifugal force and angulation angle exhibited a linear relationship (Figure 7). As the centrifugal force increased, the angulation angle also increased. One of the factors that defined performance was centripetal acceleration, which had a linear relationship with the angulation angle. However, there was no difference in centripetal acceleration from class to class. It is certain that the heavier the disability, the harder it is to obtain an angulation angle; however, during skiing, the skier is moved passively and can obtain a larger angulation angle. In other words, the difference in trunk ROM due to different degrees of disability may be canceled if the skier has mastered the technique to obtain greater centripetal acceleration.

In a linked segment system like the human body, it is possible to move a joint even when there is no actuator (muscle or motor) in the joint to be controlled, by the “interaction forces” occurred between the connected links [27]. The contribution of interaction forces/moments is significant in high‐speed movements such as pitching [28] and running [29], and many studies have been conducted on their cooperative action with muscle moments. The passive movement of the trunk observed in this study indicates that the interaction force was used to compensate for the impairment of muscles. The use of compensatory interaction forces like this is unique to para‐athletes, and it would be worth investigating in other para‐sports as well. This finding is important in the context of research on para‐sports in general as well as in the context of sit‐skiing.

**Figure 8:**
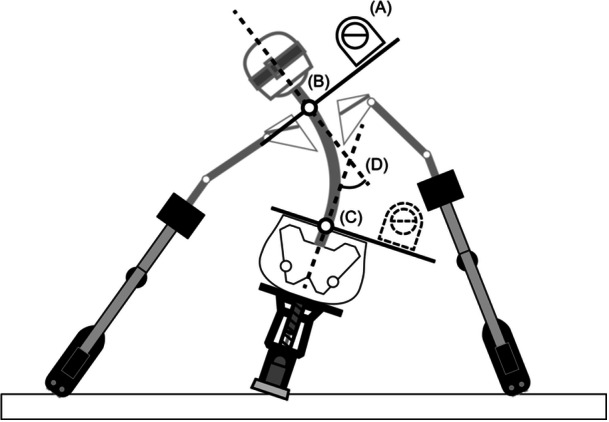
A schematic image of the proposed method for measuring the angulation angle on land. (A) The inclinometer. (B and C) The lower end of C7 vertebrae and the intersection of the upper end of the bucket seat and the spine, respectively. (D) The angulation angle to be measured. The angulation angle is obtained from the difference in inclination at Points B and C. It can be measured from the image without using an inclinometer.

By examining the ROM on the snow, it is possible to evaluate the level of a player's turn performance. This could be one of the key points for classifiers to check a skier's performance. Regarding this suggestion, we can say that it is not a good idea to evaluate board tilt in the Board Test. This is because angulation during a turn is created by the tilt of the pelvis and the tilt of the upper body. To evaluate the maximum angulation angle that a sit‐skier can take during a turn if he/she can master the best technique, it is better to measure angulation rather than board tilt even on land. One possible way to measure the maximum angulation angle is to measure the angulation angle when the sit‐skier sits on a sit‐ski on a flat surface and bends his/her trunk laterally with upper limbs and outriggers (Figure 8). The feature of this method is that it can measure the passive ROM (flexibility) of the thoracic and lumbar spinal regions, while considering the effect of the bucket seat on the ROM of the trunk. In addition, it can avoid the measurement under unstable conditions such as Test 6, since the stability of the body is important for the measurement of ROM [30]. The angle measured using this method is clearly larger than the Board Test result and closer to the value on snow. For reference, the average values for the ten athletes in this study were 27° for the angulation angle of the right turn on snow, 18° for the board tilt when bending to the left in the Board Test, and 25° for the angulation angle when bending to the left in this method. However, it is important in the assessment of the ROM of trunk to distinguish between the effects of limitations due to impairment and age, because the ROM of the spine is affected by age [31]. The effect of age might be eliminated by taking the difference between active and passive ROM, because both are affected by age. From this perspective, it may be necessary to add a test item that measures active ROM, such as measuring the angulation angle without upper limbs and outriggers in the settings of Figure 8. Further study is needed on modifying the impairment test items.

Regarding the left–right difference in turning on snow (shown in the Section 3), scoliosis may be a factor. Although this is undoubtedly a factor in the left–right difference in turning, the degree of scoliosis varies from athlete to athlete; therefore, we do not consider it necessary to evaluate the state of scoliosis in the classification test. However, we cannot rule out the possibility that the degree of scoliosis affects turning performance.

Previous research on hand cycling athletes without upper‐limb impairments has shown that trunk strength exhibits nonsignificant weak to moderate correlations with time, suggesting that trunk flexor strength does not significantly impact performance [32]. This study indicated that the classification system should focus on other determinants that affect performance. It is the same with this study, the finding that active ROM in the Board Test had minimal impact on ROM in snow conditions is a result that should be considered in future classification systems. However, it is essential to align with the fundamental principle of Paralympic classification, which aims to minimize the impact of eligible impairment types on competition outcomes. Success in Paralympic sports should be primarily determined by factors such as talent, training volume and quality, and psychological factors, rather than the severity of impairment [2, 4]. Therefore, when developing a new item for the current impairment test, it is crucial to ensure that performance enhancements through technique mastery are reliably secured.

A limitation of this study is the small sample size, which posed difficulties for hypothesis testing. The competitive population of alpine sit skiers is very small, and it is difficult to evaluate a large number of skiers with the same skill and disability level in a single country. Nevertheless, in order to progress toward evidence‐based classification, it is crucial to collect and examine data. This study employed descriptive statistics to present valuable insights in this context. Measurement of international athletes is needed in the future. The findings of this study are based on measurement data from a snow field. Compared to results obtained in a laboratory setting, the results suggest low reproducibility, which is another limitation of this study, and must be taken into account when interpreting the results. However, the conclusions are considered to be robust, as the average values of multiple turns are used and the main findings are based on results with clear differences.

## 5 Perspective

Despite IPC's recommendation for evidence‐based classification, there has been a lack of research on para‐alpine areas to provide evidence for classification. The results of this study imply that the current impairment tests are not a factor that influences the turn performance on snow. The difference in the ROM of the trunk due to different degrees of disability may be canceled if the skier has mastered the technique to obtain greater centripetal acceleration. The results of MRI and the ROM of the trunk in the Board Test demonstrated the validity of the impairment tests. This study is the first step toward evidence‐based classification of the para‐alpine sit‐skiing category. The fact that the ROM of the trunk on snow is not related to the degree of disability should be considered when considering classifications in the future. We propose measuring the angulation angle on a sit‐ski while holding an outrigger.

## Author Contributions

**Yusuke Ishige:** conceptualization, data curation, formal analysis, methodology, writing – original draft, writing – review and editing. **Yuki Inaba:** conceptualization, visualization, writing – review and editing. **Noriko Hakamada:** data curation, funding acquisition, investigation, project administration, writing – review and editing. **Akio Kobayashi:** conceptualization, resources, supervision, writing – review and editing. **Shinsuke Yoshioka:** conceptualization, formal analysis, methodology, software, supervision, visualization, writing – review and editing. All authors have read and approved the final version of the manuscript corresponding author had full access to all of the data in this study and takes complete responsibility for the integrity of the data and the accuracy of the data analysis.

## Ethics Statement

The study design was approved by the appropriate Ethics Review Board (Approval No. H30‐0047).

## Consent

The study participants provided informed consent.

## Conflicts of Interest

The authors declare no conflicts of interest.

## Transparency Statement

The lead author Yusuke Ishige affirms that this manuscript is an honest, accurate, and transparent account of the study being reported; that no important aspects of the study have been omitted; and that any discrepancies from the study as planned (and, if relevant, registered) have been explained.

## Data Availability

The data supporting the findings of this study are available upon request from the corresponding author. The data are not publicly available due to privacy or ethical restrictions.
